# Effect of High-Temperature Hydrothermal Treatment on the Cellulose Derived from the *Buxus* Plant

**DOI:** 10.3390/polym14102053

**Published:** 2022-05-18

**Authors:** Jijuan Zhang, Hongfei Huo, Lei Zhang, Yang Yang, Hongchen Li, Yi Ren, Zhongfeng Zhang

**Affiliations:** 1 College of Furniture and Art Design, Central South University of Forestry and Technology, Changsha 410004, China; t20050729@csuft.edu.cn (J.Z.); 20211200362@csuft.edu.cn (H.H.); 20210100065@csuft.edu.cn (Y.Y.); lhc1871388213@sina.com (H.L.); 20191200257@csuft.edu.cn (Y.R.); 2 Green Furniture Engineering Technology Research Center, National Forestry and Grassland Administration, Changsha 410004, China; 3 Dongyang Furniture Institute, Dongyang 322100, China; dczhangl@sina.com

**Keywords:** *Buxus*, cellulose, hydrothermal treatment, purification methods, pyrolysis

## Abstract

Cellulose has attracted considerable attention as the most promising potential candidate raw material for the production of bio-based polymeric materials. In the last decade, significant progress has been made in the production of biopolymers based on different cellulose forms. In this study, cellulose was obtained in an innovative and environmentally friendly way, using boxwood powder. Crude cellulose was obtained by treating *Buxus* powder with an ethanol–acetic acid–water mixture. Refined cellulose was then obtained by treatment with an acidic sodium hypochlorite solution and alkaline hydrogen peroxide solution. The novel chemistry of cellulose prepared by this method promises to be not only green, but also highly desirable, because of its lower emissions and low cost. It is crucial for the future of the global polymer industry. The refined cellulose was subjected to a high-temperature hydrothermal treatment under two temperatures and time conditions, with temperature gradients of 120, 140, and 160 °C, and time gradients of 1, 2, and 3 h. The samples were subjected to infrared and thermogravimetric analyses. The cellulose undergoes dehydration and thermal degradation reactions during the heat treatment process, and the thermal stability of the residual is enhanced, compared with that of virgin cellulose. Between 120 and 140 °C, the hydroxyl and hypomethyl groups on the surface of cellulose are shed. Groups in the amorphous region of the polymer are the first to be shed. The dehydration reaction reduces the number of free hydroxyl groups on the surface of the cellulose molecules. The dehydration reaction was accelerated by an increase in temperature. Between 140 and 160 °C, the β-(1,4)-glycosidic bond begins to slowly break and some furans are generated. The structure of cellulose undergoes reorganization during thermal treatment. The thermal stability of the modified material is greater than that of untreated cellulose.

## 1. Introduction

*Buxus* is a common evergreen shrub or small tree. *Buxus* branchlets are densely quadrangular, single leaf opposite, leathery, with a lightless or shiny leaf surface, neutral, light-preferring structures. They prefer warm, semi-humid climates, are fertile and moist neutral to slightly acidic soil, have low cold resistance, are tolerant to drought and barrenness, and exhibit some environmental resistance and adaptability. There are approximately 70 species of this genus distributed worldwide in Asia, Europe, and Africa. Approximately 17 species and several subspecies and varieties are known to exist in China, distributed throughout the country, mainly small-leaved *Buxus*, big-leaved *Buxus*, and Yunnan *Buxus*. Solid tight *Buxus* can be used for carving, musical instrument fabrication, and other applications. Its roots, branches, and leaves can also be used as medicinal herbs. *Buxus* not only has a role in sand control and soil conservation, but also a unique function in improving the ecosystem and maintaining the ecological balance. The *Buxus* cell wall contains a large amount of cellulose and is a natural renewable biomass resource. The rational use of *Buxus* resources results in large economic and ecological benefits. As a very large green resource, cellulose has been widely used in the food, medical, and chemical industries, in paper making, the formulation of ceramics, textiles, and perforated construction materials, and in coating formulations and other fields, owing to its good biocompatibility, low density, high strength, high crystallinity, and high hydrophilicity, among other excellent properties; it also has broad application prospects. Developing more sustainable processes for a greener and bio-based future is the current global goal. This has led to research aimed at developing bio-based polymers to address environmental concerns and reduce the current dependence on fossil resources. In 2021, 299 million tons of petroleum-based polymers were produced; with an average annual growth rate of about 4%, the demand is further increasing. Therefore, it is important to find alternatives to petroleum-based polymers. The production of oil-based polymers will be hampered by limited resources and rising raw material prices, which will pave the way for the use of alternative raw materials based on renewable feedstocks. The development of biopolymer-based materials made from renewable resources is, thus, an active area of research that is attracting increasing scientific and industrial attention. Each year, the share of bio-based polymers in the total polymer production is growing faster than the total production. First-generation bio-based polymers rely on the synthesis of building blocks (monomers) from renewable resources, including lignocellulosic biomass (starch and cellulose), fatty acids, and organic waste. As the most abundant monomer resource, the global production of lignocellulose accounts for 210.7 × 10^6^ tons of plant material per year; this does not compete with the food supply. Cellulose is one of the main components of lignocellulosic biomass and, together with lignin and hemicellulose, accounts for 35–50% of biomass. It is the strongest candidate to replace petroleum-based polymers because of its abundant and eco-friendly properties, such as renewability, biocompatibility, and biodegradability. Cellulose is a promising feedstock for the production of chemicals and cellulose-derived monomers. A variety of monomers have been obtained from cellulose through convenient catalytic processes that have potential applications in the production of biopolymers for various industries. In as early as 2013, the cellulose-based sustainable biopolymer production capacity accounted for 61.8% of the total production of biobased structural polymers, and the production is increasing year by year. In recent decades, impressive progress has been made in biobased biodegradable polymer materials based on bio(nano)polymer composites, composite films, and multifunctional composites. Cellulose-based composites are the focus of the current trends in environmentally friendly composites. The use of cellulose, cellulose nanoparticles, and cellulose derivatives as one of the (nano)composite phases, and their wide availability, have been of increasing interest in the last few years, due to their excellent mechanical properties, such as reinforcing ability, light weight, low filler loading requirements, and biodegradability. They can also be used as (nano)fillers or matrices in polymeric bio(nano)composites. However, the production of biopolymers from cellulose requires rigorous pretreatment of lignocellulose to separate it from lignin, and the subsequent biopolymer production requires deconstruction, derivatization or nanoparticle formation. Therefore, exploring methods for efficient lignocellulose extraction has become a hot research topic for efficient cellulose utilization [1,2,3,4,5,6,7,8,9,10].

Cellulose is a large polysaccharide composed of glucose connected by β-(1,4)-glycosidic bonds, with a high degree of polymerization and high average molecular weight. Morphologically, cellulose can be divided into crystalline and amorphous regions. The crystalline region contains a repeat unit of two glucose molecules connected in a 180° helix along the chain extension direction to form cellobiose. Extraction from bamboo leaves with benzene–ethanol, followed by treatment of the extracted cellulose with sodium chlorite and potassium hydroxide, generates nanocellulose. High-intensity ultrasonic irradiation has been used. The degradation of cellulose at 380 °C/40 MPa/0.12–0.48 s has been studied. Hydrolysis of cellulose with polymerization degrees of 13–100 afforded materials that, upon hydrolysis, afforded oligosaccharides with polymerization degrees of 2–12. Cellulose can be degraded to glucose, fructose, 5-HMF, and other compounds in high-temperature water, even without adding a catalyst. At present, researchers believe that cellulose is first degraded to oligosaccharides, such as cellulose hexasaccharide, cellulose pentasaccharide, cellulose tetrasaccharide, and cellulose trisaccharide, etc. Then, these oligosaccharides are degraded to glucose, which is dehydrated to 1,6-anhydride-bonded anhydrous glucose, and also to fructose through an isomerization reaction. Both glucose and fructose can be decomposed to erythrose, ethanol aldehyde, dihydroxyacetone, and glyceraldehyde; glyceraldehyde can be converted to dihydroxyacetone; dihydroxyacetone and glyceraldehyde can be dehydrated to acetone aldehyde; and acetone aldehyde, erythrose and ethanol aldehyde can be further decomposed to form smaller molecules. The hydrolytic conversion of cellulose in high-temperature hydrothermal treatment is mainly influenced by the reaction temperature and reaction time. Studies on high-temperature liquid water show that when the temperature of high-temperature liquid water changes from 200 °C to 300 °C, the ionic product of high-temperature liquid water increases by three orders of magnitude, which greatly enhances the hydrolysis reaction, while the dielectric constant decreases with the increase in temperature, which favors the dissolution of the glucose oligomers produced by hydrolysis, and the resulting increase in temperature can destroy the oxygen bonding network in cellulose. Some scholars have found that when the temperature of high-temperature liquid water rises to 300 °C, the breakage of oxygen bonds in cellulose can even transform crystalline cellulose into amorphous cellulose, revealing that the change in the nature of high-temperature liquid water, caused by the change in temperature, is an important cause of the fiber degradation reaction. However, there are few studies on the hydrothermal treatment of cellulose at 100–200 °C, and research in this area will provide help for the efficient utilization of cellulose [11,12,13,14,15,16,17,18,19,20,21,22].

Cellulose has been extracted from *Buxus* using purification and bleaching processes to remove hemicellulose and lignin, and to obtain a high-purity cellulose. The cellulose was submitted to high temperature and high pressure using water as a heat transfer medium. The effect of the high-temperature hydrothermal treatment on the structure of the *Buxus* cellulose has been evaluated and compared with the impact of a similar process of lower temperature. This high-temperature process may have potential for generating materials that permit the utilization of *Buxus* cellulose for the development of value-added products.

## 2. Experimental Section

Materials and chemicals: *Buxus* cubes with a prism length of 2 cm were employed. After cleaning and drying, we selected wood blocks without a defect split. A small wood bundle was placed into a high-speed crusher to obtain a powder. We screened 40–100-mesh *Buxus* powders for the experiments. Anhydrous ethanol was obtained from Tianjin Damao Chemical Reagent Factory. Glacial acetic acid was obtained from Tianjin Fuchen Chemical Reagent Co. Sodium chlorite, sodium hydroxide, and hydrogen peroxide were purchased from Aladdin Reagent Co. All above reagents were of analytical purity. Potassium bromide obtained from Aladdin Reagents Ltd. was spectrally pure.

Pretreatment with an ethanol–acetic acid–water mixture: Firstly, 4 g of the *Buxus* powder was weighted in a 50 mL polytetrafluoroethylene bottle with an electronic precision balance. Following this, 40 mL of the ethanol–acetic acid–water mixture (mass fraction of ethanol of 45% and mass fraction of acetic acid of 1%) was poured into the bottle and loaded into a reaction kettle. We set the heating temperature to 200 °C and holding time to 1 h. The solution was then cooled to room temperature. Subsequently, the mixture was poured out, filtered, washed with 70% (mass concentration) ethanol, washed with secondary distilled water, and dried at 105 ± 3 °C to obtain a crude cellulose product.

Acidic sodium chlorite solution treatment: Firstly, 10 g of the crude cellulose of the *Buxus* was weighted in a 500 mL three-mouth flask. We added 250 mL of a mixed solution of 30 mL /L acetic acid and sodium chlorite (volume fraction) in a solid–liquid ratio of 1:25, processed at 75 °C for 60 min, followed by filtering, washing, and drying, which yielded the preliminary refined cellulose.

Alkaline hydrogen peroxide solution treatment: After the optimal process for sodium chlorite bleaching was determined, the cellulose required for the subsequent process was prepared in batches, according to the optimized conditions. The refinement was then continued using an alkaline hydrogen peroxide bleaching process to further improve the purity of cellulose. A section of 10 g of the refined cellulose was weighed and added to a 500 mL three-neck flask. Following this, 250 mL of the alkaline hydrogen peroxide bleaching solution was added at a solid-to-liquid ratio of 1:25, treated at 70 °C for 70 min, and then filtered, washed, and dried to obtain the refined cellulose.

**Figure 1:**
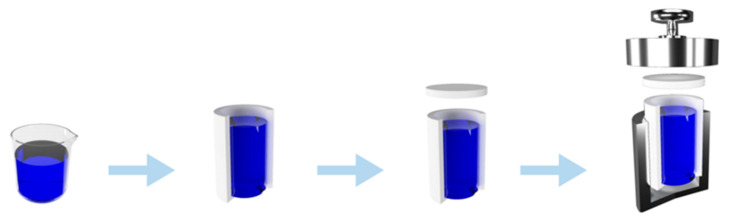
Schematic diagram of the hydrothermal treatment process of boxwood cellulose.

High-temperature hydrothermal cellulose modification stage: The refined cellulose was placed in a small high-temperature hydrothermal treatment instrument and controlled by electric heating, to hydrothermal treatment temperatures of 120, 140, and 160 °C. The hydrothermal treatment time for each treatment temperature was set to 1, 2, and 3 h. The bath ratio was 1:10. The hydrothermal treatment time is employed to reach the set temperature after the holding time. At this time, the evaporation rates of water and steam coalescences are equal (dynamic equilibrium state of water and gas). The internal pressure of the container was set to the corresponding temperature of the saturated vapor pressure. After the hydrothermal treatment, the system was cooled to room temperature and the treated cellulose was removed. The process is shown in Figure 1.

Analytical methods: Scanning electron microscopy was carried out on a field emission scanning electron microscope after the samples were sputtered with gold. A Fourier-transform infrared spectrometer was used to analyze different samples. The scanning wave number range was set to 4000–400 cm^−1^, the cumulative number of scans was set to 32, and the resolution was set to 4 cm^−1^. Then, 0.2 g potassium bromide pressed tablets were used to collect the background. The sample pressed tablets were added at 0.002 g and the error was controlled within 0.0001 g. The charge volume of the thermogravimetric analysis was within 50 mg, the carrier gas was nitrogen with a flow rate of 50 mL·min^−1^, and the temperature rise program was set to 30 K·min^−1^ from 30 to 600 °C.

## 3. Results and Discussion

### 3.1. Morphological Analysis

**Figure 2:**
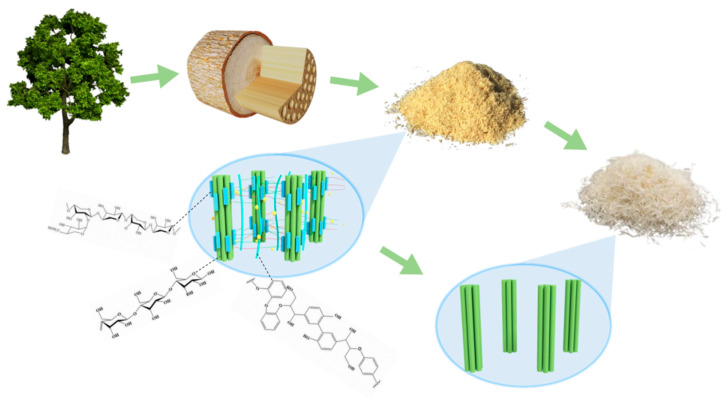
Schematic diagram of the preparation of boxwood cellulose.

As shown in Figure 2, with the preparation, the color of the *Buxus* powder gradually became lighter and turned white after the acidic sodium chlorite and alkaline hydrogen peroxide treatment, indicating that lignin was removed and cellulose was further purified at these two stages.

**Figure 3:**
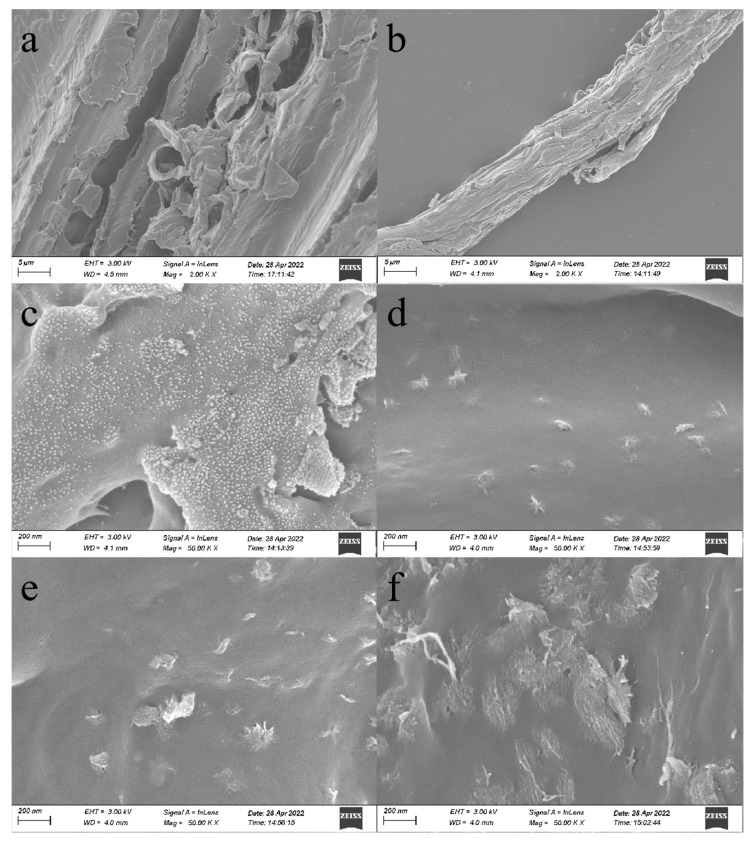
SEM morphological analysis: (**a**) raw wood powder magnified 2000 times; (**b**) cellulose magnified 2000 times; (**c**) cellulose magnified 50,000 times; (**d**) cellulose magnified 50,000 times (120 °C, 1 h); (**e**) cellulose magnified 50,000 times (120 °C, 2 h); (**f**) cellulose magnified 50,000 times (120 °C, 3 h).

**Figure 4:**
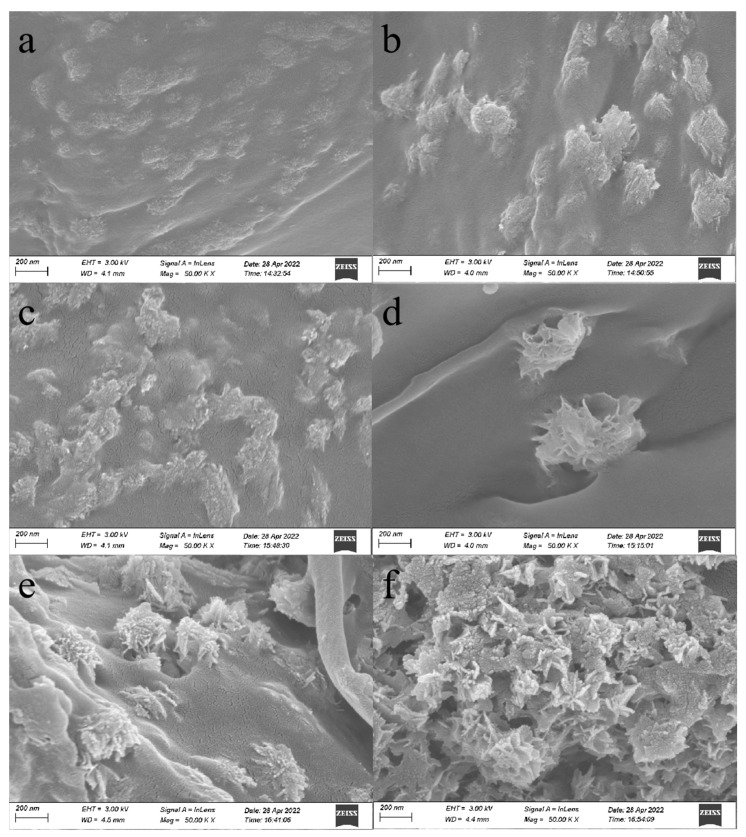
SEM morphological analysis: (**a**) cellulose magnified 50,000 times (140 °C, 1 h); (**b**) cellulose magnified 50,000 times (140 °C, 2 h); (**c**) cellulose magnified 50,000 times (140 °C, 3 h); (**d**) cellulose magnified 50,000 times (160 °C, 1 h); (**e**) cellulose magnified 50,000 times (160 °C, 2 h); (**f**) cellulose magnified 50,000 times (160 °C, 3 h).

From Figure 3 and Figure 4, it is possible to notice that the surface of the boxwood powder can be observed in the direction of the texture of the fiber, in addition to obvious lamellar structures and cavities. The overall surface of the refined cellulose fiber is smooth, and the magnification to 50,000 times reveals a large number of spherical attachments on the surface, which are various groups of polysaccharides, which can explain the hydrophilicity of cellulose. When the reaction temperature reached 120 °C, the long chains of cellulose macromolecules were broken, resulting in the sample surface becoming rough, loose, and porous; the number of irregular pits on the cell wall surface increased, the depth increased, the skirt-like structure appeared, and the spherical attachments on the cell wall surface disappeared and were replaced by some clusters. The reason for this is that the hydrated hydrogen ions, generated by the self-ionization of water molecules, break the glycosidic bonds on the cellulose macromolecule chains, and the non-crystalline region of cellulose is hydrolyzed, which produces oligomers that are hydrolyzed into different monomers, and these monomers then produce 1,6-dehydrated glucose, erythrulose, furfural complexes, etc., through dehydration, ring-opening reactions, and C-C bond-breaking reactions. These products then undergo intermolecular dehydration or aldol condensation reactions, in order to induce further polymerization or polycondensation reactions to form polymers. At the same time, the polymers undergo aromatization reactions, in which the hydroxyl groups in the monomer molecules are dehydrated to form C-O, and the intermolecular dehydration of monomer molecules, or the keto-enol interconversion isomerization of the already dehydrated monomer molecules, forms C-C, which corresponds to the peaks at 1655 cm^−1^ and 1425 cm^−1^ in the IR spectra. That is, the oligosaccharides or monosaccharides decompose or dehydrate to form aromatized molecules, which subsequently condense to form compounds. When the reaction temperature reached 160 °C and the time was 1 h, a small amount of microsphere structures of different sizes formed on the surface of cellulose, and, with the increase in reaction time, microsphere structures started to appear in a large area, and the size also gradually became larger, reaching 200 nm. The microsphere structures became smoother with the increase in residence time on the surface, and the homogeneity and dispersion also became better. From this morphological change occurring at temperatures between 120 and 160 °C, it can be inferred that the temperature at which cellulose started to generate furan-like substances was around 150 °C.

### 3.2. FTIR Analysis

**Figure 5:**
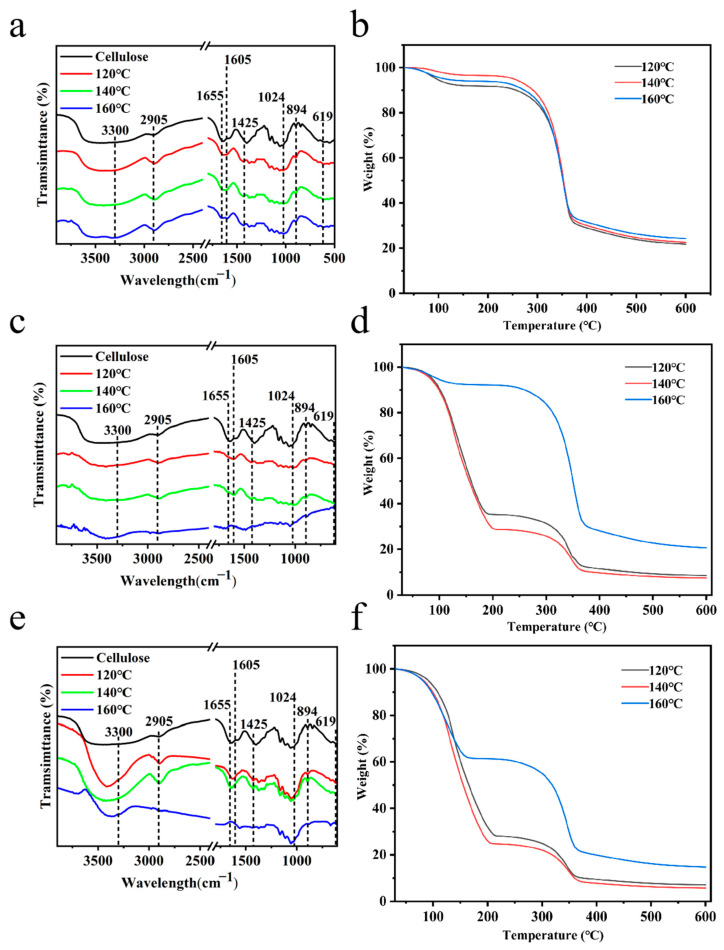
FTIR curves of samples obtained at different temperatures for different treatment durations ((**a**): 1 h, (**c**): 2 h, and (**e**): 3 h); thermogravimetric curves of samples obtained at different temperatures for different treatment durations ((**b**): 1 h, (**d**): 2 h, and (**f**): 3 h).

By analyzing the IR spectrum of the prepared cellulose, it can be observed in Figure 5 that the broad absorption band near wave number 3300 cm^−1^ is the stretching vibration peak of -OH; the absorption peak near wave number 2905 cm^−1^ is the C-H stretching in -CH_3_ and the -CH_2_ vibration peak; the absorption peak near wave number 1655 cm^−1^ is the C-O vibration peak; the absorption peak near wave number 1605 cm^−1^ is the bending vibration peak of cellulose C-C; the absorption band near wave number 1425 cm^−1^ is the crystalline region of cellulose and the bending vibration peak of cellulose -CH_2_; the strong absorption peak near wave number 1024 cm^−1^ is the stretching vibration peak of C-O in the fiber backbone, which is the characteristic absorption peak of cellulose; the stronger absorption peak near wave number 894 cm^−1^ is the bending deformation vibration peak of the β-(1,4)-glycosidic bond to connect the sugar units; and the absorption peak near wave number 619 cm^−1^ is the out-of-plane bending vibration peak of O-H, which is the vibration due to the presence of cellulose [23,24,25,26,27].

By analyzing the infrared spectra of cellulose samples under different temperature conditions, with the same treatment time, it can be found that the absorption peak at 3300 cm^−1^ has weakened or disappeared, and that the absorption peak at 1700 cm^−1^ has enhanced or appeared, which indicates that the dehydration reaction occurred during hydrothermal carbonization, and the C-O bond may be a carbonyl group or a carboxyl group. Both are formed by the dehydration of hydroxyl groups. The absorption peak near wave number 2905 cm^−1^ tends to decrease with the increase in temperature, indicating that the hypomethyl group in the amorphous region of cellulose is the first to be shed; meanwhile, the enhancement or appearance of the absorption peak at 1605 cm^−1^ confirms the existence of an aromatic ring, thus indicating that the aromatization process occurs during hydrothermal carbonization. The absorption peak near wave number 1425 cm^−1^ was significantly weakened, indicating that the heat treatment degraded the methylene groups in the crystalline region of cellulose in the chemical structure, resulting in a decrease in cellulose content; the absorption peaks near wave number 890 cm^−1^ did not change significantly at 120 °C and 140 °C, and the absorption peak was slightly weakened at 160 °C, indicating that β-(1,4)-glycosidic bond breakage occurred between 140 °C and 160 °C. The absorption peaks near the cellulose characteristic peak wave numbers 1065 cm^−1^ and 619 cm^−1^ tended to weaken, confirming the degradation of the cellulose structure. By analyzing the IR spectra of cellulose samples under different time conditions at the same treatment temperature, it can be found that the peaks with more obvious changes are the absorption peaks near wave number 2920 cm^−1^, decreasing with time, indicating that the degradation of hypomethyl groups is more affected by time; the absorption peaks near wave number 890 cm^−1^ have no obvious increase with time, indicating that the β-(1,4)-glycosidic bond is broken more slowly [28,29,30,31].

### 3.3. Thermogravimetric Analysis

The first stage, from 30 to 180 °C, includes the precipitation of water and bound water, and the pre-pyrolysis stage, which is also known as the slight weight loss stage and is the transition stage of pyrolysis. As the temperature increases, the state of matter starts to change to a molten state during gradual thermal decomposition. The second stage (180 to 580 °C) is the main stage of pyrolysis. The relative weight loss rate was calculated separately for the second stage of the pyrolysis reaction to avoid the effect of errors in water content on the overall structural analysis. As shown in Table 1, the relative weight loss was more than 70%, which was mainly due to the violent decomposition caused by the cleavage of C-O and C-C bonds in the cellulose structure and the formation of a hydrocarbon structure, resulting in the generation of small-molecule gases and large-molecule condensable gases. It also includes the decomposition of residual lignin and tar carbonization. With the increase in treatment time, the comparison of the samples at the same temperature shows that the relative weight loss rates of the samples exhibited a decreasing trend, and that the maximum weight loss rate temperature increased. When the treatment time was the same, with the increase in treatment temperature, the relative weight loss rates of the samples exhibited an increasing trend and the maximum weight loss rate temperature decreased. The thermal stability of cellulose is enhanced [32,33].

## 4. Conclusions

(1) Cellulose undergoes a dehydration reaction and thermal degradation during the heat treatment process. Between 120 °C and 140 °C, the hydroxyl group and hypomethyl group on the surface of cellulose are shed, and the amorphous region is the first to be shed; the dehydration reaction occurs between cellulose molecules, so the number of free hydroxyl groups on the surface of the cellulose molecules is reduced, and the dehydration reaction is accelerated with the increase in temperature. The hydrolysis of cellulose polysaccharides produces oligomers, i.e., oligosaccharides or monosaccharides, that decompose or dehydrate to form aromatized molecules, and the aromatized molecules then condense to form compounds. Between 140 °C and 160 °C, the β-(1,4)-glycosidic bond appears to break slowly and some furans are formed. The temperature variation had a large effect on the overall structural stability of cellulose, and the effect caused by time variation was not significant.
(2) The main pyrolysis stage of cellulose was in the range of 180 to 580 °C, in which cellulose decomposed to produce a small-molecule gas and large-molecule condensable gas. The relative weight loss rate reached above 70%. The weight loss rate increased with the increase in heat treatment temperature. With the increase in treatment time, the weight loss peak temperature decreased, the relative weight loss rate decreased, and the weight loss peak temperature increased. The thermal stability of cellulose was, therefore, enhanced.

## Figures and Tables

**Table 1:** Thermogravimetric relative weight loss rate calculation for the second stage.

High Temperature Hydrothemal Treatment Method	Initial Weight (mg)	First Stage Weight Loss (mg)	First Stage Remaining (mg)	Second StageWeight Loss (mg)	Second Stage Remaining (mg)	Second Stage Relative Weight Loss Rate (%)
120 °C, 1 h	12. 8635	1.0480	11.8155	8.9711	2.8444	8.9711
120 °C, 2 h	11.5720	0.3940	11.1780	8.5233	2.6547	8.5233
120 °C, 3 h	8.8292	0.5287	8.3005	6.1386	2.1619	6.1386
140 °C, 1 h	30.9825	20.0611	10.9214	8.2474	2.674	8.2474
140 °C, 2 h	40.8035	29.1543	11.6492	8.5577	3.0915	8.5577
140 °C, 3 h	14.0785	1.1126	12.9659	9.9724	2.9935	9.9724
160 °C, 1 h	52.1125	37.7879	14.3246	10.5711	3.7535	10.5711
160 °C, 2 h	41.4759	31.2786	10.1973	7.7947	2. 4026	7.7947
160 °C, 3 h	22.8505	8.8163	14.0342	10.5617	3.4725	10.5617

## Data Availability

Data is contained within the manuscript and Supplementary Material.

## Supplementary Materials

The following supporting information can be downloaded at: https://www.mdpi.com/article/10.3390/polym14102053/s1, DTG: The charge volume of the thermogravimetric analysis was within 50 mg, the carrier gas was nitrogen with a flow rate of 50 mL·min^−1^, and the temperature rise program was set to 30 K·min^−1^ from 30 to 600 °C. FTIR: A Fourier-transform infrared spectrometer was used to analyze different samples. The scanning wave number range was set to 4000–400 cm^−1^, the cumulative number of scans was set to 32, and the resolution was set to 4 cm^−1^. 0.2 g potassium bromide pressed tablets were used to collect the background. The sample pressed tablets were added at 0.002 g and the error was controlled within 0.0001 g. SEM: Scanning electron microscopy was carried out on a fieldemission scanning electron microscope after the samples were sputtered with gold.
