# Supplementary material for: Effect of High-Temperature Hydrothermal Treatment on the Cellulose Derived from the Buxus Plant
Source: Polymers (Basel). 2022 May 18;14(10):2053. doi: 10.3390/polym14102053 (PMC9143544; doi:10.3390/polym14102053)
Supplement: Supplementary file 1 [file polymers-14-02053-s001.zip › DTG/2-XX.pdf]

!2-XX

Author: METTLER TOLEDO
Date: 2021/11/8
Database: STARe Default DB V16.30: METTLER

Evaluation: !2-XX, 08.11.2021 20:06:03

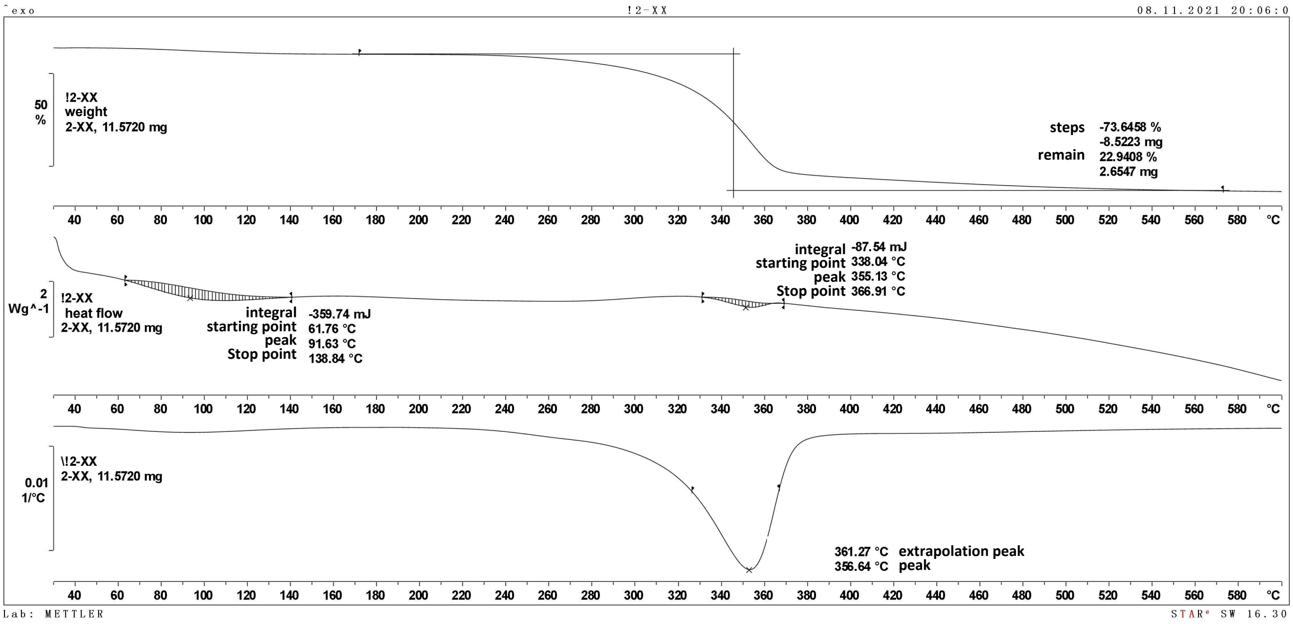

Curve: **!2-XX, 08.11.2021 20:06:03**

Sample: 2-XX, 11.5720 mg

Sample Holder: Alumina 70ul
Ceramic

Method: Central South
Forest-Lignin
dt 1.00 s
[1] 30.0..600.0 °C, 30.00 K/min, N2 50.0 ml/min
Sync Enable

Module: TGA/DSC 3+ LF/1100/1024, 12.07.2021 13:32:05

User: METTLER

Curve: **!2-XX, 08.11.2021 20:06:03**

Sample: 2-XX, 11.5720 mg

Sample Holder: Alumina 70ul
Ceramic

Method: Central South
Forest-Lignin
dt 1.00 s
[1] 30.0..600.0 °C, 30.00 K/min, N2 50.0 ml/min
Sync Enable
