# Supplementary material for: Effect of High-Temperature Hydrothermal Treatment on the Cellulose Derived from the Buxus Plant
Source: Polymers (Basel). 2022 May 18;14(10):2053. doi: 10.3390/polym14102053 (PMC9143544; doi:10.3390/polym14102053)
Supplement: Supplementary file 1 [file polymers-14-02053-s001.zip › DTG/6-XX.pdf]

Author: METTLER TOLEDO
Date: 2021/11/9
Database: STARe Default DB V16.30: METTLER

# Evaluation:

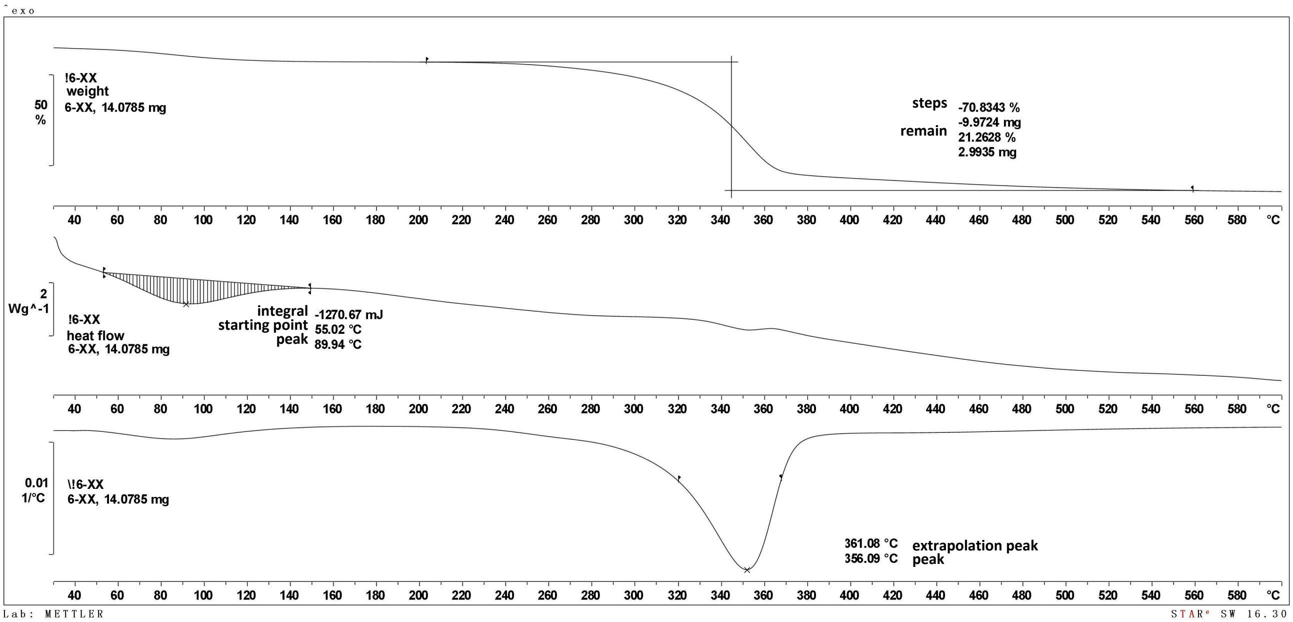


**Curve:** **!6-XX**

- **Sample:** 6-XX, 14.0785 mg
- **Sample Holder:** Alumina 70ul
  Ceramic
- **Method:** Central South
  Forest-Lignin
  dt 1.00 s
  [1] 30.0..600.0 °C, 30.00 K/min, N2 50.0 ml/min
  **Sync Enable**
- **Module:** TGA/DSC 3+ LF/1100/1024, 12.07.2021 13:32:05
- **User:** METTLER

---

**Curve:** **!6-XX**

- **Sample:** 6-XX, 14.0785 mg
- **Sample Holder:** Alumina 70ul
  Ceramic
- **Method:** Central South
  Forest-Lignin
  dt 1.00 s
  [1] 30.0..600.0 °C, 30.00 K/min, N2 50.0 ml/min
  **Sync Enable**
