# Supplementary material for: Effect of High-Temperature Hydrothermal Treatment on the Cellulose Derived from the Buxus Plant
Source: Polymers (Basel). 2022 May 18;14(10):2053. doi: 10.3390/polym14102053 (PMC9143544; doi:10.3390/polym14102053)
Supplement: Supplementary file 1 [file polymers-14-02053-s001.zip › DTG/7-XX.pdf]

!7-XX

Author: METTLER TOLEDO
Date: 2021/11/9
Database: STARe Default DB V16.30: METTLER

Evaluation: !7-XX, 09.11.2021 10:58:00

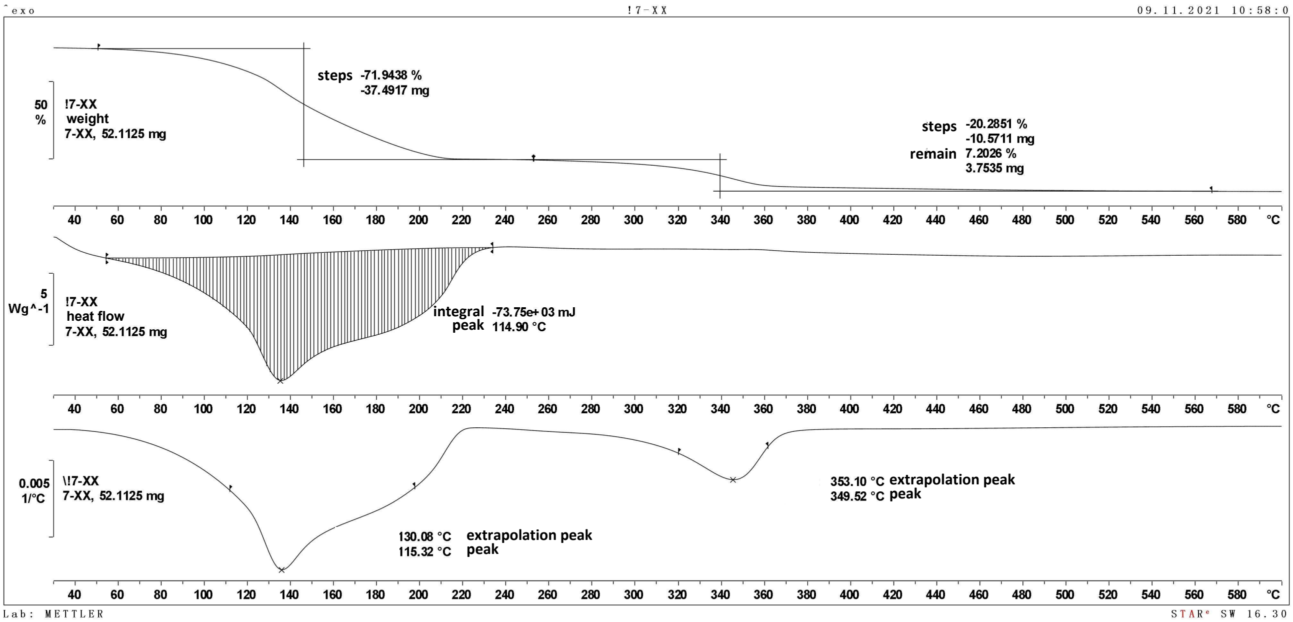

Curve: **!7-XX, 09.11.2021 10:58:00**

Sample: 7-XX, 52.1125 mg
Sample Holder: Alumina 70ul
Ceramic
Method: Central South
Forest-Lignin
dt 1.00 s
[1] 30.0..600.0 °C, 30.00 K/min, N2 50.0 ml/min
Sync Enable
Module: TGA/DSC 3+ LF/1100/1024, 12.07.2021 13:32:05
User: METTLER

Curve: **!7-XX, 09.11.2021 10:58:00**

Sample: 7-XX, 52.1125 mg
Sample Holder: Alumina 70ul
Ceramic
Method: Central South
Forest-Lignin
dt 1.00 s
[1] 30.0..600.0 °C, 30.00 K/min, N2 50.0 ml/min
Sync Enable
