# Supplementary material for: Effect of High-Temperature Hydrothermal Treatment on the Cellulose Derived from the Buxus Plant
Source: Polymers (Basel). 2022 May 18;14(10):2053. doi: 10.3390/polym14102053 (PMC9143544; doi:10.3390/polym14102053)
Supplement: Supplementary file 1 [file polymers-14-02053-s001.zip › DTG/8-XX.pdf]

Author: METTLER TOLEDO
Date: 2021/11/9
Database: STARe Default DB V16.30: METTLER

Evaluation:

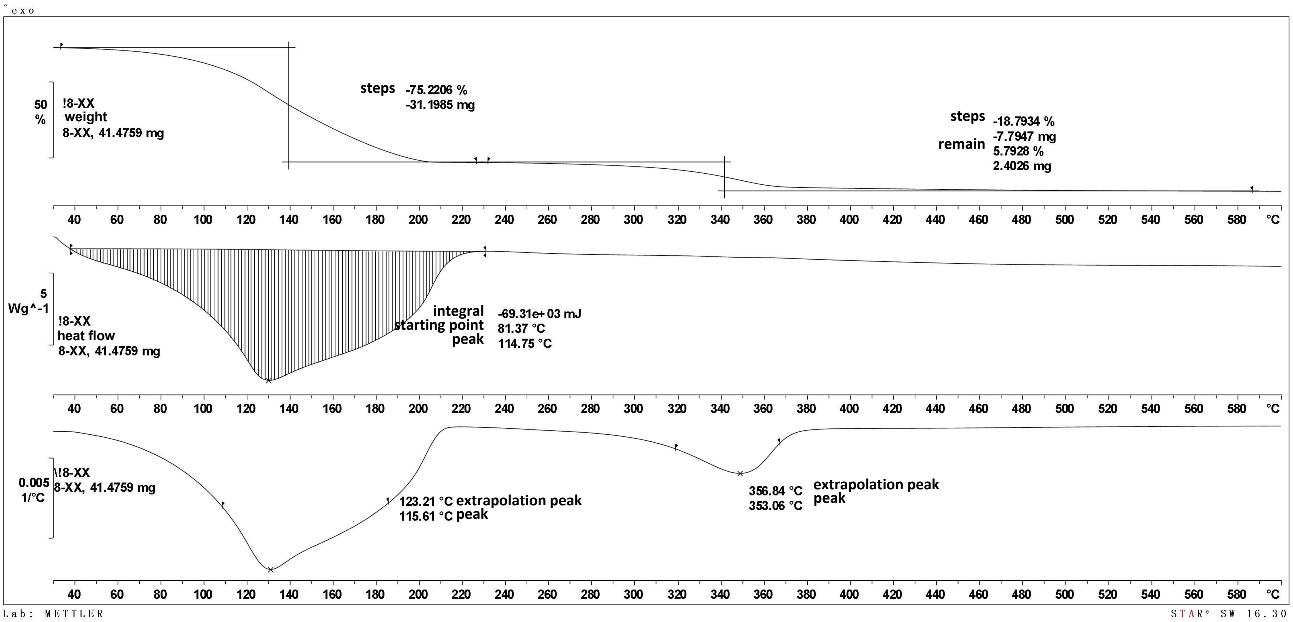

**Curve:**	**!8-XX**
**Sample:**	8-XX, 41.4759 mg
**Sample Holder:**	Alumina 70ul<br>Ceramic
**Method:**	Central South<br>Forest-Lignin<br>dt 1.00 s<br>[1] 30.0..600.0 °C, 30.00 K/min, N2 50.0 ml/min<br>**Sync Enable**
**Module:**	TGA/DSC 3+ LF/1100/1024, 12.07.2021 13:32:05
**User:**	METTLER

**Curve:**	**!8-XX**
**Sample:**	8-XX, 41.4759 mg
**Sample Holder:**	Alumina 70ul<br>Ceramic
**Method:**	Central South<br>Forest-Lignin<br>dt 1.00 s<br>[1] 30.0..600.0 °C, 30.00 K/min, N2 50.0 ml/min<br>**Sync Enable**
